# Use of Mapping and Spatial and Space-Time Modeling Approaches in Operational Control of *Aedes aegypti* and Dengue

**DOI:** 10.1371/journal.pntd.0000411

**Published:** 2009-04-28

**Authors:** Lars Eisen, Saul Lozano-Fuentes

**Affiliations:** Department of Microbiology, Immunology and Pathology, Colorado State University, Fort Collins, Colorado, United States of America; University of Oxford, United Kingdom

## Abstract

The aims of this review paper are to 1) provide an overview of how mapping and spatial and space-time modeling approaches have been used to date to visualize and analyze mosquito vector and epidemiologic data for dengue; and 2) discuss the potential for these approaches to be included as routine activities in operational vector and dengue control programs. Geographical information system (GIS) software are becoming more user-friendly and now are complemented by free mapping software that provide access to satellite imagery and basic feature-making tools and have the capacity to generate static maps as well as dynamic time-series maps. Our challenge is now to move beyond the research arena by transferring mapping and GIS technologies and spatial statistical analysis techniques in user-friendly packages to operational vector and dengue control programs. This will enable control programs to, for example, generate risk maps for exposure to dengue virus, develop Priority Area Classifications for vector control, and explore socioeconomic associations with dengue risk.

## Purpose of the Review

Numerous reviews have broadly addressed the use of geographical information system (GIS), remote sensing (RS), and spatial and space-time modeling approaches in the field of vector-borne diseases [1]–[4]. However, the critically important issue of the potential for such technologies and methodologies to be used for operational prevention, surveillance, and control of neglected tropical vector-borne diseases has not received the attention it deserves. Adaptation of mapping and modeling solutions for use in resource-constrained disease-endemic environments must be made part of the new frontier in vector-borne disease research. Our review focuses specifically on dengue, which is caused by mosquito-borne dengue viruses, and aims to 1) provide an overview of how mapping and spatial and space-time modeling approaches have been used to date to visualize and analyze mosquito vector and epidemiologic data; and 2) discuss the potential for these approaches to be included as routine activities in operational vector and dengue control programs.

## Introduction to Mapping and Spatial and Space-Time Modeling Approaches to Facilitate Operational Control of *Aedes aegypti* and Dengue

Dengue and other diseases caused by arboviruses maintained in mosquito–human transmission cycles are characterized by dramatic outbreaks that may overwhelm public health capacity for outbreak control and supportive patient care [5]. In the case of dengue, where a vaccine against the virus is still lacking, vector control program activities during outbreaks focus on reducing mosquito vector populations to levels where dengue virus transmission no longer is sustainable and the role of the mosquito is reduced to that of a nuisance biter [6]. However, controlling the primary dengue virus vector, *Aedes aegypti*, has proven a difficult undertaking in the modern urban landscape. This, in part, is due to the biology of the mosquito. *Ae. aegypti* exploits a wide variety of containers that are found in domestic habitats as larval development sites, including containers ranging in size from bottles and cans to large water storage tanks [7]. Uncontrolled urban growth, which often is accompanied by a lack of piped water or unreliable water supplies (thus promoting water storage), and the proliferation of non-degradable trash containers in today's throwaway society combine to provide an ample supply of larval development sites and makes it difficult to effectively control *Ae. aegypti*.

Success stories for the control of *Ae. aegypti* have in recent years often come from atypical situations where a few easily identified and treated container types account for the vast majority of mosquito production; e.g., wells and water storage tanks in rural areas of Vietnam [8]. As a further complication, the female mosquito is adapted to use the indoor environment where she preferentially feeds and rests, and may also lay her eggs if suitable containers are available [9],[10]. This creates a situation where 1) labor-intensive and costly house-to-house indoor application of insecticides may be required to effectively disrupt a dengue outbreak; and 2) targeting of surveillance and control efforts to high-risk areas can help to overcome the logistical challenges related to achieving early outbreak detection and effective outbreak control.

Increasingly user-friendly GIS software and other emerging mapping technologies, such as Google Earth and Microsoft Virtual Earth, provide new opportunities to visualize spatial and space-time patterns for entomological and epidemiological data, and to generate risk models for vector and dengue virus exposure [11]–[13]. Locations where vector data were collected or where human disease cases occurred can be determined with a global positioning system (GPS) receiver or directly from a high-quality image of the environment. Field data collection where GPS receivers are used together with handheld personal data assistants can facilitate rapid transfer of data to an electronic database and subsequent use of a GIS or other mapping platform for data visualization and analysis [14]–[17]. Key benefits of using GIS-based approaches include the capacity to link different types of information for a given spatial location or area (e.g., land cover, climate factors, socioeconomic variables, and entomological and epidemiological data), potential for spatial statistical analysis, and development of spatial databases that can be used for a wide range of public health programs [12]. Another practical application is ongoing mapping of dengue case locations in relation to spatial coverage of implemented vector control [18].

## Use of Mosquito Vector Data versus Dengue Case Data in Mapping and Modeling

The relative value of mapping and spatial modeling based on entomological versus epidemiological data differs among vector-borne diseases [13]. In the case of dengue, there are good reasons to focus on epidemiological data rather than mosquito vector data. **First**, the human-biting female is notoriously difficult to collect, which has led to an emphasis on surveillance of the immature larval and pupal stages [19]. The value of using data for immatures to assess spatial patterns of dengue risk has been brought into question. Although some studies have reported that larval indices are predictive of spatial risk for dengue virus transmission [20]–[22], others have failed to find significant associations between immature indices or abundances and spatial patterns of dengue incidence [23]–[26]. The use of data for females from oviposition traps shows some promise [27]; however, there is a critical need for improved methodology to determine epidemiologically significant measures of the indoor abundance of host-seeking and resting females in order to enhance the usefulness of entomological data for spatial modeling of dengue risk.

**Second**, spatial abundance patterns of *Ae. aegypti* are strongly influenced by the presence and abundance of containers that serve as larval development sites. This presents a major obstacle for the development of fine-scale predictive models for vector abundance because it is unlikely that even very fine-scale aerial photography or RS imagery will be useful in detecting the plethora of containers that are exploited as larval development sites [28]. **Third**, correlates of vegetation (e.g., Normalized Difference Vegetation Index or greenness derived from RS imagery) may be of some use [29], but mainly in situations where the primary mosquito-producing containers are rain-filled, rather than filled by humans, and shading therefore may prevent containers from drying out. Climate variables may be informative across large geographical areas, for example at the scale of Puerto Rico, where rainfall patterns and access to naturally water-filled containers differ between the southern, drier and northern, wetter parts of the island [30], but are less likely to be of predictive value within the confines of a single city. **Fourth**, the risk of dengue outbreaks is influenced not only by the abundance of *Ae. aegypti* females but also by dengue virus serotype-specific herd immunity (against dengue virus serotypes 1–4) among the human population [31],[32].

### Operational Implications

Based on the above considerations, mapping and spatial modeling based on mosquito presence or abundance data [27], [33]–[41] should be viewed as only representing potential dengue risk. Further, operational use of mosquito abundance thresholds to signal the risk of dengue outbreaks is hampered by the fact that 1) these thresholds will fluctuate with the level of serotype-specific herd immunity among the human population; and 2) up-to-date information for serotype-specific herd immunity rarely is available in operational settings [32]. In contrast, the presence of a dengue case demonstrates human contact with an infected vector (unless infection resulted from transfusion of dengue virus-infected blood). Mapping and spatial modeling based on epidemiological data thus represents actual rather than potential dengue risk. Drawbacks to the use of dengue case data include the occurrence of asymptomatic infections [42], difficulty in conclusively determining virus exposure sites (although indoor environments, especially homes, are considered key locations for dengue virus exposure [43],[44]), and the potential for long delays in laboratory confirmation of suspected dengue cases. These shortcomings need to be taken into account when basing operational decisions on maps or spatial models that were developed based on dengue case data.

## Mapping Approaches: Maps as Tools for Delivery of Vector and Dengue Information

**Figure 1:**
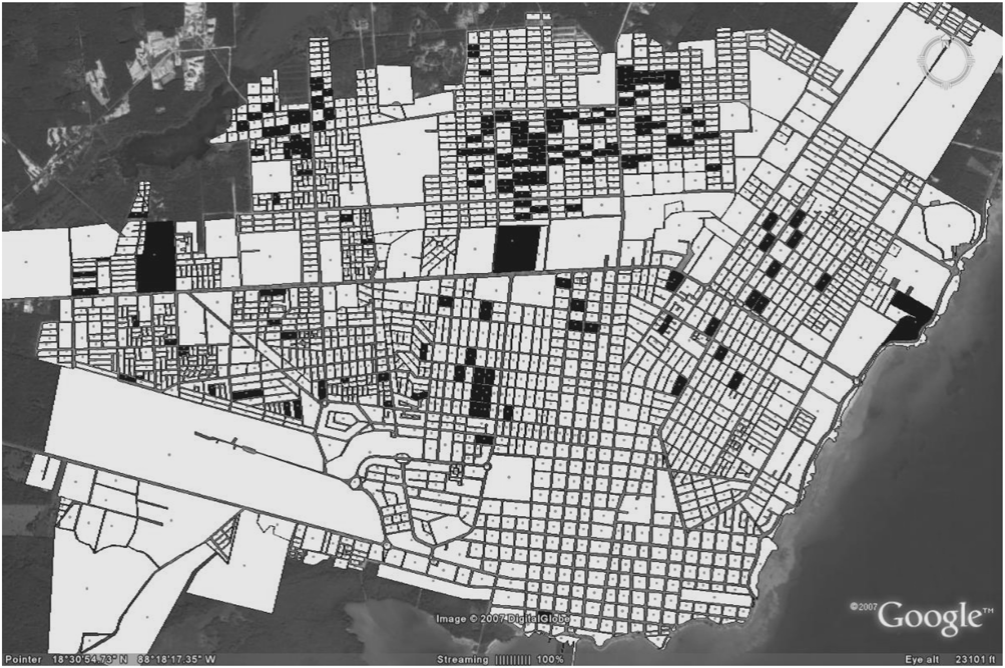
Distribution of City Blocks with Dengue Cases (Filled) versus City Blocks without Dengue Cases (Unfilled) in 2006 in Chetumal, Quintana Roo, Mexico. This map was published previously by Lozano-Fuentes et al. (2008) in the *Bulletin of the World Health Organization* [11].

Maps are powerful tools for information delivery. Consider first the statement “dengue cases were concentrated to the northern part of the city”. This provides a general idea of where disease cases were most common but does not necessarily provoke further interest. By complementing the statement “dengue cases were concentrated to the northern part of the city” with a map showing dengue case locations (Figure 1), a visual stimulus is added to effectively capture the imagination of the audience. One common initial response to the map will be to determine where cases occur relative to the viewer's own place of residence. Another will be to start thinking about why cases cluster to the north, or even why they occur within certain blocks but not others; this will draw on the viewer's own knowledge of the city and likely generate ideas to explain the observed pattern.

GIS-based dengue occurrence or risk maps are increasingly generated in dengue-endemic regions of Southeast Asia and the Americas at spatial scales ranging from individual villages, neighborhoods, towns, or cities [17], [20], [22], [29], [38], [45]–[61] to districts [62]–[64] and countries [16], [21], [65]–[71]. The use of GIS software for this purpose is now complemented by other emerging mapping technologies such as Google Earth, which provides free access to satellite imagery and has a basic capacity to generate both static dengue occurrence or risk maps [11] and dynamic time-series maps that show how the spatial distribution of dengue cases changes over time. For an in-depth discussion of the benefits and drawbacks of using these mapping software programs relative to GIS software, see Lozano-Fuentes et al. [11].

### Operational Implications

Maps showing mosquito vector data (presence or abundance patterns), epidemiological data (dengue case locations or dengue incidence patterns), or coverage of implemented vector control are useful tools both internally in a vector/dengue control program, where they can be used to guide and assess the progress of operational activities, and for disseminating information to outside parties. For example, maps can be helpful to alert the public of areas within a city with an especially high risk of dengue virus exposure, or to inform local policy makers with jurisdiction over the vector/dengue control program budget. The maps can be distributed through multiple information dissemination routes, including reproduction in newspapers or pamphlets and posting on internet sites. The emergence of new mapping technologies provides another intriguing route for the distribution of maps. For instance, Keyhole Markup Language (KML) files generated in Google Earth can be downloaded from Web sites or sent as e-mail attachments and then viewed, including the capability to zoom in to areas of special interest, by the recipient. The only requirement for viewing the KML file, which for example can contain the dengue occurrence map shown in Figure 1, is to first download the free Google Earth software.

## Mapping Approaches: Dengue Case-Driven “Detect-and-Respond” Vector Control Emergency Response

It may be tempting to use new mapping capacity to implement “fire-fighting” or “detect-and-respond” style strategies where vector control teams are sent out in response to clinically diagnosed or laboratory-confirmed dengue cases. Control approaches based on detection of and response to dengue cases or clusters can, however, be questioned because they ignore virus transmission from persons with mild, undifferentiated fevers that are not recognized as being caused by dengue viruses [72] and may not be effective when there are long time lags for laboratory confirmation of suspected cases. Below we present scenarios for making judicious use of mapping capacity to guide emergency vector control response activities during dengue outbreaks.

### Operational Implications

Numerous studies have reported the presence of dengue virus–infected *Ae. aegypti* females in the homes of dengue patients [43], [61], [73]–[75], which demonstrates the value of indoor application of insecticides in these homes to destroy infected mosquitoes and thus prevent visiting persons from being bitten by infected mosquitoes and later potentially starting new transmission foci in other areas. Mapping capacity can aid with the operational logistics of directing vector control teams to these homes. However, one intriguing question with direct bearing on operational vector and dengue control activities is what additional efforts should be undertaken in order to prevent local virus spread. The fact that dengue cases commonly cluster in space and space-time [21], [22], [29], [46], [49], [51], [53]–[55],[61],[71],[76] indicates that expansion of vector control activities to include a perimeter around a known case location is a rational approach as long as diagnostic confirmation of cases is timely. In this response scenario, basic mapping capability allows for effective visualization of both case locations and response perimeters [14]. One recent study from Thailand demonstrated that implementation of integrated vector control within 100 m around dengue case homes resulted in decreased exposure to dengue virus compared to untreated areas [59].

This type of “detect-and-respond” approach will, however, not address dispersal by infected humans beyond the selected control perimeter and likely will fail during an outbreak when numerous new transmission foci appear over short time periods [29],[51]. The best solution to the operational conundrum of how to most cost-effectively implement vector control emergency response is perhaps a two-tier strategy. This would entail 1) a response to dengue cases or clusters during periods with low transmission activity; and 2) a switch during periods of high transmission activity to a strategy focusing on the entire vector control program area and using Priority Area Classification (PAC) to determine the order in which sub-areas are treated (see the next section for more detail). Research is urgently needed to determine epidemiological surveillance thresholds (e.g., based on weekly reported cases per 1,000 persons) to trigger a switch between these two vector control strategies.

## Mapping Approaches: Development of PAC Schemes for Emergency Vector Control

During an outbreak, dengue cases often spread rapidly throughout a city [29],[51],[71] and may become so numerous and widespread that vector control response capacity is overwhelmed. Barrera et al. [45] noted that 70% of all reported dengue cases in Maracay, Venezuela, during 1993–1998 occurred within 55 of the city's neighborhoods (covering only 35% of the city's total area) and proposed that these neighborhoods should be the highest priority areas for vector control. Similarly, 7 years of retrospective epidemiological data were used to develop a three-level dengue transmission risk classification for census tracts in the city of Belo Horizonte, Brazil [48]. Entomological indices were used in a similar manner to identify key areas for vector control in the city of Nova Iguaçu, Brazil [34].

### Operational Implications

PAC for emergency vector control during dengue outbreaks is perhaps the best example where a dengue case and incidence mapping approach is directly useful in guiding operational activities. During dengue outbreaks, the most rational vector control strategy is to ignore the locations of individual cases and instead activate a PAC-based emergency response scenario where high-risk areas are prioritized and treated before areas with lower risk. In addition to emergency vector control during dengue outbreaks, PACs also can be used to guide spatial implementation of proactive vector control efforts or vector surveillance schemes.

## Spatial Modeling Approaches: Environmental and Socioeconomic Associations with the Risk of Dengue Virus Exposure

Increasing access to spatially explicit environmental data (e.g., land cover, vegetation indices, and climate data) and socioeconomic data (e.g., presence of piped water, reliability of water supply, income and housing characteristics) provides new opportunities to determine factors that are predictive of the risk of dengue virus exposure [47],[49],[52],[55],[56],[70]. For example, a regression modeling approach can be used to generate models where environmental and/or socioeconomic factors (independent variables) extracted from GIS- or RS-based data layers are used to predict dengue incidence (dependent variable) [22],[48]. Because the independent variables are available as spatial data layers, it also is possible to generate a spatial surface (map) for projected dengue risk based on the model equation. Furthermore, Bayesian statistical analysis techniques are emerging [77],[78] and these are now beginning to be applied in dengue risk modeling [64],[79],[80].

### Operational Implications

The spatial modeling approaches outlined above likely will become commonplace as increasing numbers of countries develop high quality demographic and socioeconomic spatial data. However, development and validation of spatially predictive dengue risk models is needed before they can be considered useful for operational vector and dengue control. The extent to which a dengue risk model developed and validated in one city is applicable also to other cities in the same country or region also needs to be explored.

## Space-Time Modeling Approaches: Determination of Dengue Outbreak Dynamics

Understanding, and ultimately being able to predict, the spatiotemporal dynamics of dengue outbreaks or epidemics at spatial scales ranging from cities to countries and continents is critical to our ability to prevent and control the disease. GIS software and improved analysis techniques provide opportunities to study and model spatiotemporal dynamics of dengue outbreaks [29],[46],[50],[51],[54],[57],[71],[81]. Indeed, studies on dengue outbreak dynamics are increasingly using statistical analysis techniques to explore dengue case clustering in space (e.g., determination of measures of spatial autocorrelation or use of spatial scan statistics) or space-time (e.g., the Knox test) [21], [22], [29], [38], [46], [49], [51], [53]–[56],[58],[71],[76]. This commonly reveals that dengue cases are clustered in space or space-time. We expect to see vigorous growth in this field with the continual emergence of new analysis techniques, e.g. Bayesian space-time analysis techniques [82]. Benefits of Bayesian approaches include a more rigorous accounting of uncertainty compared to models based on frequency probability.

### Operational Implications

**Figure 2:**
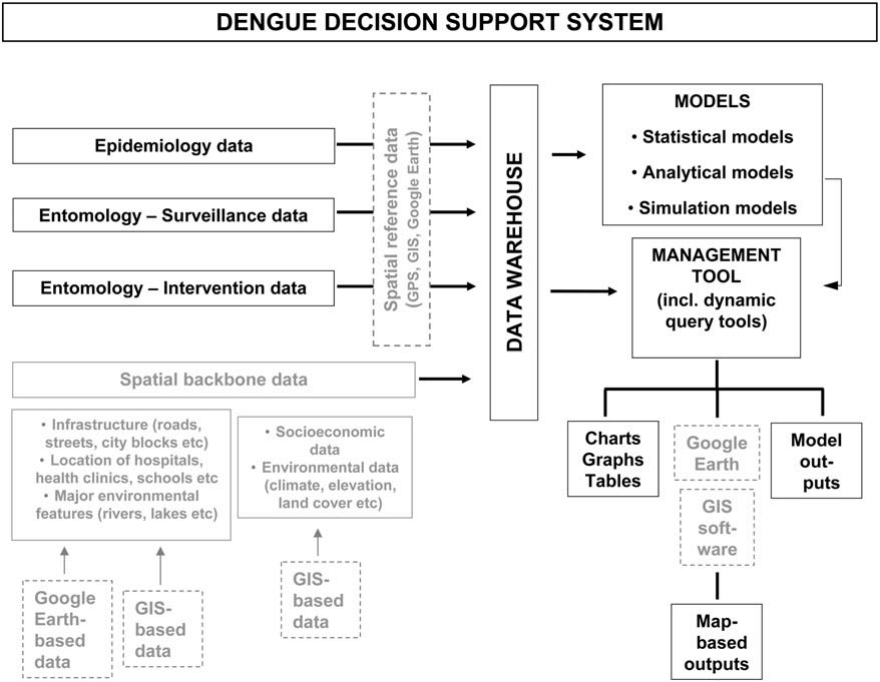
Potential Use of Google Earth and Geographical Information System (GIS) Software in a Basic Dengue Decision Support System Framework. Adapted from a schematic published previously by Lozano-Fuentes et al. (2008) in the *Bulletin of the World Health Organization* [11].

One key challenge for this emerging field is to move from the research arena to practical applications that can enhance operational vector and dengue control. For example, analysis of dengue outbreaks at national scales may reveal spatiotemporal trends that are repeated in successive outbreaks. This can then be exploited by a national vector control program to implement a nationwide resource allocation scheme that stays one step ahead of the spatiotemporal dynamic of a future outbreak. Further, capacity for basic time-series mapping needs to be transferred to local vector and dengue control programs. The importance of developing local mapping capacity cannot be overstated: this will empower local control programs to include spatial and spatiotemporal disease case mapping as a routine activity and make it part of the control program decision-making process (Figure 2). As noted previously, emerging user-friendly and free mapping technology such as Google Earth can now be used to produce and disseminate dynamic space-time disease case maps at a minimal cost. We also see the potential for including spatial cluster analysis in routine operational epidemiological surveillance. Key issues to address before implementing cluster analysis as a routine tool to help guide operational vector and dengue control include 1) selection of appropriate analysis techniques; 2) the definition of what constitutes a cluster of dengue cases; 3) the length of the time period used in the cluster analysis; 4) the nature and spatial extent of the response activity triggered by the detection of a case cluster; and 5) evaluation of the efficacy of strategies guided by this method.

## Future Directions in the Mapping and Modeling of Vector and Dengue Data

In addition to the research needs outlined in the text, we would like to highlight the following future directions:

- Perhaps the most important future direction is to make mapping and spatial modeling technologies and methodologies readily available to the public health community in dengue-endemic countries. This should include the development of user-friendly computer-based systems with the ability to generate map outputs and to run basic spatial and space-time statistical analyses from data in locally maintained epidemiological databases. We see several benefits from this approach, including 1) production of useful map outputs; 2) potential for determination of factors underlying observed disease risk patterns; and 3) technology use as a focal point to bring together governmental and non-governmental agencies in a common effort to meet the threat of dengue.
- The emergence of an inexpensive diagnostic test to determine serotype-specific dengue virus exposure would open the door for a new generation of serotype-specific dengue mapping and spatial modeling efforts. This would increase the value of such risk assessment approaches to inform operational vector and dengue control.
- When a vaccine against dengue virus becomes available, mapping of dengue case and incidence patterns can help to focus public health–driven immunization campaigns toward areas with high transmission risk. We also see the potential for initiating routine mapping of vaccine delivery to keep track of immunization coverage, for example by census area of residence. This would further increase the effectiveness of targeted immunization campaigns by enabling the combination of spatial information on the historical risk of dengue virus transmission with knowledge of current immunization coverage.

## Methods

The literature search included the use of multiple online databases (Biological Abstracts, ISI Web of Knowledge, Ovid MEDLINE) and retrieval through Colorado State University's electronic interlibrary loan system of additional relevant publications discovered through perusal of publications and their reference lists. Key word combinations in the online literature searches included the following: dengue and GIS, dengue and remote sensing, dengue and mapping, dengue and modeling, dengue and spatial, dengue and space-time, *Aedes* and GIS, *Aedes* and remote sensing, *Aedes* and mapping, *Aedes* and modeling, *Aedes* and spatial, *Aedes* and space-time, vector-borne and GIS, vector-borne and remote sensing, vector-borne and mapping, vector-borne and modeling, vector-borne and spatial, and vector-borne and space-time. Papers with direct relevance to the core topic (mapping and spatial modeling of dengue risk) were included in the review. For supporting information, we included selected representative references.

**Learning Points**

1. GIS software are becoming more user-friendly and will be increasingly used as operational tools for mapping and spatial analysis as more countries develop GIS-based data for infrastructure and demographic and socioeconomic factors.
2. Free mapping software such as Google Earth are emerging as a powerful complement to GIS software for mapping purposes by providing access to satellite imagery, basic feature-making tools, and the capacity to generate both static maps and dynamic time-series maps to visualize spatiotemporal disease outbreak dynamics.
3. Our challenge is now to move beyond the research arena and to transfer mapping and GIS technologies and spatial statistical analysis techniques in user-friendly packages (e.g., in the form of a dengue decision support system) to operational vector and dengue control programs.
4. Mapping and spatial modeling can aid operational vector and dengue control by enabling local control programs to, for example, generate static and dynamic dengue occurrence or risk maps, develop Priority Area Classifications for vector control, and explore socioeconomic associations with dengue risk.
5. Space-time analysis will help us to understand, and ultimately predict, spatiotemporal dynamics of dengue outbreaks at spatial scales ranging from cities to countries and continents.

**Key Papers in the Field**

1. Indaratna K, Hutubessy R, Chupraphawan S, Sukapurana C, Tao J, et al. (1998) Application of geographical information systems to co-analysis of disease and economic resources: dengue and malaria in Thailand. Southeast Asian J Trop Med Public Health 29: 669–684. ***Country-scale analysis of spatial dengue and malaria risk patterns in relation to socioeconomic factors.***
2. Morrison AC, Getis A, Santiago M, Rigau-Perez JG, Reiter P (1998) Exploratory space-time analysis of reported dengue cases during an outbreak in Florida, Puerto Rico, 1991–1992. Am J Trop Med Hyg 58: 287–298. ***Uses space-time analysis techniques to study a dengue outbreak.***
3. Ai-leen GT, Song RJ (2000) The use of GIS in ovitrap monitoring for dengue control in Singapore. Dengue Bull 24: 110–116. ***Describes the use of GIS in a mosquito vector monitoring program.***
4. Barrera R, Delgado N, Jiménez M, Villalobos I, Romero I (2000) Estratificación de una ciudad hiperendémica en dengue hemorrágico. Rev Panam Salud Publica 8: 225–233. ***Stratifies a city by incidence of dengue hemorrhagic fever and proposes a basic priority area classification for vector control.***
5. Lozano-Fuentes S, Elizondo-Quiroga D, Farfan-Ale JA, Loroño-Pino MA, Garcia-Rejon J, et al. (2008) Use of Google Earth to strengthen public health capacity and facilitate management of vector-borne diseases in resource-poor environments. Bull WHO 86: 718–725. ***Uses Google Earth to develop municipal infrastructure maps and to generate dengue risk maps.***
